# B Cells Adapt Their Nuclear Morphology to Organize the Immune Synapse and Facilitate Antigen Extraction

**DOI:** 10.3389/fimmu.2021.801164

**Published:** 2022-02-09

**Authors:** Romina Ulloa, Oreste Corrales, Fernanda Cabrera-Reyes, Jorge Jara-Wilde, Juan José Saez, Christopher Rivas, Jonathan Lagos, Steffen Härtel, Clara Quiroga, María-Isabel Yuseff, Jheimmy Diaz-Muñoz

**Affiliations:** ^1^ Departamento de Biología Celular y Molecular, Facultad de Ciencias Biológicas, Pontificia Universidad Católica de Chile, Santiago, Chile; ^2^ Laboratory for Scientific Image Analysis SCIAN-Lab, Programa de Biología Integrativa, Instituto de Ciencias Biomédicas ICBM, Facultad de Medicina, Universidad de Chile and Biomedical Neuroscience Institute BNI, Facultad de Medicina, Universidad de Chile, Santiago, Chile; ^3^ Centro de Informática Médica y Telemedicina CIMT, Facultad de Medicina, Universidad de Chile and Centro Nacional en Sistemas de Información en Salud CENS, Santiago, Chile; ^4^ División de Enfermedades Cardiovasculares, Facultad de Medicina, Pontificia Universidad Católica de Chile, Santiago, Chile; ^5^ Advanced Center for Chronic Diseases (ACCDiS), Universidad de Chile and Pontificia Universidad Católica de Chile, Santiago, Chile

**Keywords:** B cells, nuclear morphology, immune synapse, actin cytoskeleton, Nesprin-1, Sun-1

## Abstract

Upon interaction with immobilized antigens, B cells form an immune synapse where actin remodeling and re-positioning of the microtubule-organizing center (MTOC) together with lysosomes can facilitate antigen extraction. B cells have restricted cytoplasmic space, mainly occupied by a large nucleus, yet the role of nuclear morphology in the formation of the immune synapse has not been addressed. Here we show that upon activation, B cells re-orientate and adapt the size of their nuclear groove facing the immune synapse, where the MTOC sits, and lysosomes accumulate. Silencing the nuclear envelope proteins Nesprin-1 and Sun-1 impairs nuclear reorientation towards the synapse and leads to defects in actin organization. Consequently, B cells are unable to internalize the BCR after antigen activation. Nesprin-1 and Sun-1-silenced B cells also fail to accumulate the tethering factor Exo70 at the center of the synaptic membrane and display defective lysosome positioning, impairing efficient antigen extraction at the immune synapse. Thus, changes in nuclear morphology and positioning emerge as critical regulatory steps to coordinate B cell activation.

## Introduction

Efficient uptake and processing of foreign antigens by B cells is critical for their complete activation (1, 2). In lymph nodes, B cell activation is initiated by the engagement of the B cell receptor (BCR) with antigens displayed at the surface of neighboring cells, which triggers tyrosine kinase-dependent signaling cascades (3–7). BCR signaling is coupled to a rapid actin-dependent membrane spreading response, followed by an acto-myosin-dependent contraction phase, enabling B cells to concentrate antigens at the center of the immune synapse (8–11). Concomitantly, B cells re-position the MTOC to the center of the synaptic membrane, which acts as a landmark to guide the polarized recruitment of lysosomes, which upon secretion can facilitate antigen extraction (12–14). How B lymphocytes manage to position the MTOC and target lysosomes to precise domains of the immune synapse remains incompletely understood. Recently, the tethering factor Exo70, a subunit of the exocyst complex, was shown to be involved in lysosome docking at the synaptic membrane in B cells. Exo70 is associated to the MTOC in resting B cells and becomes repositioned to the synaptic membrane upon activation, helping to promote lysosome tethering and fusion (15).

B cells possess reduced cytoplasmic space, occupied mainly by their large nucleus (16), which is closely associated to the MTOC, from which it becomes uncoupled during activation (17). We thus sought to determine the impact of nuclear morphology in cell reorganization during immune synapse formation of B cells. Nuclear morphology and positioning have been shown to regulate diverse cellular functions, including signaling, gene expression (18), DNA repair or genome distribution (19–21), as well as cell shape (22) and migration (23–25). Nuclear size, form and positioning rely on the Linker of Nucleoskeleton and Cytoskeleton (LINC) complex (26–28). This complex is formed by two families of transmembrane proteins: KASH/Syne/Nesprin proteins, anchored to the outer nuclear membrane, where they are connected to cytoskeletal components; and Sun domain proteins inserted at the inner nuclear membrane, which are associated to lamins and chromatin (29). Interactions between nuclear envelope proteins and the surrounding cytoskeleton regulate nuclear positioning and cell polarity (28). For instance, Nesprin-Sun complexes directly connect actin filaments and microtubules with the nucleus, to control both nuclear and MTOC repositioning during cell migration (30). In activated T lymphocytes, Lamin A interacts with the Nesprin-Sun complex to promote MTOC repositioning towards the immune synapse (31).

Whether B lymphocytes adjust their nuclear morphology to promote immune synapse organization, has not been addressed. In this work we reveal that, upon activation with immobilized antigens, B cells re-orientate their nuclear groove, in an actin and microtubule dependent manner, towards the antigen contact site, where the immune synapse is formed. Silencing the expression of nuclear envelope proteins, Nesprin-1 or Sun-1, decreased the connection between cytoskeleton and nucleus and impaired nuclear reorientation to the immune synapse. Strikingly, these cells displayed an extremely disorganized immune synapse, characterized by decreased actin cytoskeleton levels and deficient internalization of the BCR at the center of the synapse. Additionally, Nesprin-1 and Sun-1 silenced B cells mislocated the tethering factor Exo70 and were unable to concentrate lysosomes to the center of the synaptic interface, resulting in decreased antigen extraction capacity. Thus, our results highlight how B cells adjust nuclear morphology to cytoskeleton rearrangements at the immune synapse to orchestrate lysosome recruitment and enable them to acquire their antigen extraction and processing functions.

## Materials and Methods

### Antibodies and Reagents

We used a F(ab’)2 goat anti-mouse immunoglobulin G (anti-IgG) (MP Biomedical, Santa Ana, CA) or a F(ab’)2 goat anti-mouse immunoglobulin M (Jackson Immunoresearch) as a BCR ligands, Ovalbumin (Sigma #A5503), Hoechst 33342 (Thermo Fisher Scientific #62249), Wheat germ agglutinin (WGA) Alexa Fluor® 555 Conjugate (Invitrogen #W32464) as a non-activating ligand.

The following primary antibodies were used for immunofluorescence: rabbit anti-mouse Lamin B1 (Abcam; ab16048, 1:500), rabbit anti-mouse anti-SUN1 (Abcam, ab103021), donkey anti-mouse Alexa 647 (Invitrogen #A31571), mouse anti- Nesprin1 (Invitrogen, MA5-18077 MANNES1A, 7A12), rabbit anti-Nesprin1/Syne-1 (Abcam, ab192234), rabbit anti- EXOC7 (Abcam, ab95981) rabbit-CEP55 (Abcam, ab170414), rat anti-mouse α- tubulin (Abcam; abab6160, 1:500), rat anti-mouse LAMP1 (BD Biosciences; #553792, 1:200), rabbit anti-OVA (Sigma- Aldrich; #C6534, 1:500) and F(ab’)2 goat anti-mouse immunoglobulin G (Jackson ImmunoResearch) Alexa Fluor 488. The following secondary antibodies were used: Alexa Fluor 488– conjugated goat anti-rabbit (LifeTech, 1:200), Alexa Fluor 647 and Cy3–conjugated F(ab)2 donkey anti-rat; and Cy3-conjugated F(ab)2 donkey anti-rabbit (Jackson ImmunoResearch; 1:200), Rhodamine Phalloidin (Invitrogen,#R415), 1:100) and Hoechst (Thermo Fisher Scientific, #33342), 1:1,000). For Western blot, the following antibodies were used: rabbit anti-Sun-2 (Abcam #124916, 1:100), mouse IgG1 anti-Nesprin1 (Invitrogen #MANNES1A (7A12), 1:500), rat anti- α- tubulin (Genetex, #GTX76511), 1:100), rabbit anti- γ-Tubulin (Abcam, #Ab11317, 1:1,000), mouse anti-actin (cloneC4, ImmunO, #691001), mouse Nesprin 1 mouse (invitrogen, Ma5-18077, 1:500) rabbit anti-Histone 3 (Abcam #ab1791). As secondary antibody: HRP-conjugated donkey anti-rat, anti-rabbit or anti-mouse (Jackson ImmunoResearch; 1:5,000). For cytoskeleton-disrupting drugs, we incubated 1h, used 5 µM Latrunculin A, 1μg/ml Cytochalasin D, 30 µM Nocodazole, 10 µM Taxol (paclitaxel Merck), 1 μM Suberoylanilide Hydroxamic Acid (SAHA, Cayman Chemical Company) and 70 μM Blebbistatin (Merck).

### Cell Lines and Culture

The mouse IgG+ B-lymphoma cell line IIA1.6 (32) was used. Cells were cultured in CLICK medium (RPMI 1640 with 10% fetal bovine serum, Glutamax supplemented and 1 mM sodium pyruvate, 100 µg/ml streptomycin, 100 U/ml penicillin and 0.1% β- mercaptoethanol (12). HEK 293T cells were cultured for lentiviral production in DMEM supplemented with 10% FBS and penicillin/streptomycin. Cell culture products were purchased from Life Technologies. Spleen derived primary B cells were isolated from C57BL/6 mice using a magnetic cell sorting B cell isolation kit (Miltenyi) according to the manufacturer’s instructions. Mice protocols were approved by the Institutional Scientific Ethics Committees for Animal and Environmental Care and Research Biosafety, Pontificia Universidad Católica de Chile.

### Cell Transfection

The Amaxa Cell Line Nucleo- factor Kit R (L-013 program; Lonza) was used to electroporate 5 × 10^6^ IIA1.6 B Lymphoma cells for different transfections. For Centrin-GFP plasmid transfection we used 2 µg of DNA, and cells were cultured for 16 h before functional analysis. In the case of transfection with siRNA we used Silencer^®^ Select (Ambion/Thermofisher) against Nesprin-1(Syne1): s234287 (#1), s234288 (#2); siRNA Sun1: s94911 (#1), s94912 cells (#2), these were incubated for 48 h before analysis. As a control, we used a scrambled siRNA (Qiagen) at 10nM.

### Preparation of Antigen-Coated Beads and Coverslips

Antigen-coated beads or antigen-coated dishes were prepared as previously described (13). Briefly, to prepare antigen-coated beads, ~2 x 10^7^ 3-μm latex NH2-beads (Polyscience) were activated with 8% glutaraldehyde for 4 h at room temperature. Beads were washed with cold PBS and incubated overnight at 4°C with 100 μg/mL of F(ab’)2 goat anti-mouse IgG or IgM, referred to as antigen or activating beads. WGA-coated beads were used as an irrelevant ligand that binds to N-acetyl-D-glucosamine (GlcNAc), sialic-acid-containing glycoconjugates and oligosaccharides present on the plasma membrane (33). Antigen coated coverslips were prepared by incubating 10mm coverslips with 0.1mg/ml of anti-IgG in PBS, overnight at 4°C. Dishes were washed and used immediately for activation assay.

### Activation of B Cells and Immunofluorescence

Cells were plated on poly-l-Lysine–coated glass coverslips and activated with antigen-coated beads (1:1 ratio) or on antigen coverslips for different time points in a cell culture incubator (37°C, 5% CO2) and then fixed in 4% paraformaldehyde (PFA) for 10 min at room temperature as previously described (13). Fixed cells were incubated with antibodies in PBS-0.2% BSA-0.05% Saponin and mounted (ref).

### Antigen Extraction Assay

B cells were incubated in a 1:1 ratio beads containing anti-IgG and OVA (100 μg/mL each) and plated on poly-l-Lysine coverslips at 37°C for different time points, fixed and stained for OVA. The amount of OVA remaining on the beads was calculated by establishing a fixed area around beads in contact with cells and measuring fluorescence on three-dimensional (3D) projections obtained from the sum of each plane. The percentage of antigen extracted was estimated by the percentage of fluorescence intensity lost at the beads after 0, 30 min and 1h and 2h of incubation with B cells.

### Live Imaging of the Nucleus and MTOC in B Cells

In order to measure nuclear rotation and MTOC positioning, centrin-GFP expressing B cells were stained with Hoechst and plated onto anti-IgG coated glass coverslips for different time points at 37°C, 5% CO2 and images were acquired every 2 min by epifluorescence microscopy.

### Cytoplasm, Nuclear and Perinuclear Fractionation

Subcellular fractionation was performed as previously described for (34), that we adapted to B cells. 10 x10^6^ B cells (activated for 0, 30, 60, 120 min) were lysed in buffer I (40 mM Hepes pH7.4, 120 mM KCl, 2 mM EGTA, 0.4% Glycerol, 10 mM b-glycerophosphate and protease inhibitors plus 0.4% NP-40 plus) while rotating for 30 min at 4°C. The intact nuclei were separated from the *Cytoplasm Fraction*, pelleted by centrifugation at 1,000 g for 5 min. To obtain the cytosolic fraction, the supernatant was centrifuged at 10,000 g for 10 min. The pellet of nuclei was sequentially washed with 0.1% NP-40 and Buffer I and centrifuged at 1,000 g for 5 min. The nuclear pellet was re-suspended in Buffer II (10 mM Tris-HCl pH 7.4, 1.5 mM KCl, 0.5% Triton X-100; 0.5% Deoxycholate, 2.5 mM MgCl2, with fresh 0.2M LiCl and protease inhibitors) in a ratio 1:2 v/v and rotated for 1 h at 4°C. The extract was separated by centrifugation at 2,000 g for 5 min and to obtain the *Perinuclear Fraction*, the extract was centrifuged further at 10,000 g for 10 min. The core nuclei pellet was resuspended in a buffer containing 0.34 M sucrose and centrifuged at 2,000 g for 10 min. To obtain the core *Nuclear Fraction*, nuclei were dissolved in 8 M urea, sonicated and clarified by centrifugation at 10,000 g for 10 min. Each fraction was resuspended in loading buffer 3x, boiled, and loaded onto a 10% SDS-PAGE gel

### Immunoprecipitation Assay

Immunoprecipitation was performed as previously described by (35), adapted to B cells. 10 x10^6^ B cells (in resting conditions or activated for different times point) were incubated with IP buffer (10 mM Hepes, pH 7.4, 10 mM KCl, 5 mM EDTA, 1% Triton X-100 and protease inhibitor cocktail), sonicated on ice and then centrifuged at 16,000 g for 15 min at 4°C. Lysates were then precleared with protein A agarose beads (Roche). IPs with rabbit- anti-Nesprin-1 (abcam ab192234) and non-specific rabbit IgG (4 µg antibody/500 µg of B cell lysates) were performed at 4°C overnight, followed by incubation with protein A agarose beads at 4°C for 2 h and centrifugation at 4,000 g for 10 min. Samples were washed, resuspended in loading buffer 3x, boiled, and loaded onto a 8% SDS-PAGE gel.

### BCR Cell Surface Staining

B cells, under resting conditions or after 15 min of activation with a BCR-ligand were incubated on ice and stained for the BCR by using an anti-mouse 647 antibody. Labelling was performed under non-permeabilizing conditions for 20 min, after which cells were fixed with PFA. The amount of BCR at the cell surface was calculated by establishing a fixed area around cells and measuring fluorescence on three-dimensional (3D) projections obtained from the sum of each plane.

### Cell Imaging

Confocal images were obtained with a Zeiss LSM880 Airyscan Confocal microscope with a 63X/1.4NA oil immersion lens, in 16-bit Z-stack image sets with 0.2 µm optical section and 0.07 µm x 0.07 µm pixel size. For OVA extraction assays, epifluorescence imaging was used. Z-stack images were obtained with 0.5 microns between slices. Images were acquired in an epifluorescence microscope (Nikon Ti2Eclipse) with a 60 X/1.25NA oil immersion objective.

### Image Analysis

Image processing and analysis were performed with FIJI/ImageJ software (36). 3D analysis was performed using the SCIAN-Soft software tools (https://github.com/scianlab), programmed on the IDL programming language (ITT/Harris Geospatial; Boulder, CO). Image brightness and contrast were manually adjusted for visualization purposes but not for analysis (e.g. in the case of segmentations).

#### Nuclear Groove Reorientation

**Figure 1:**
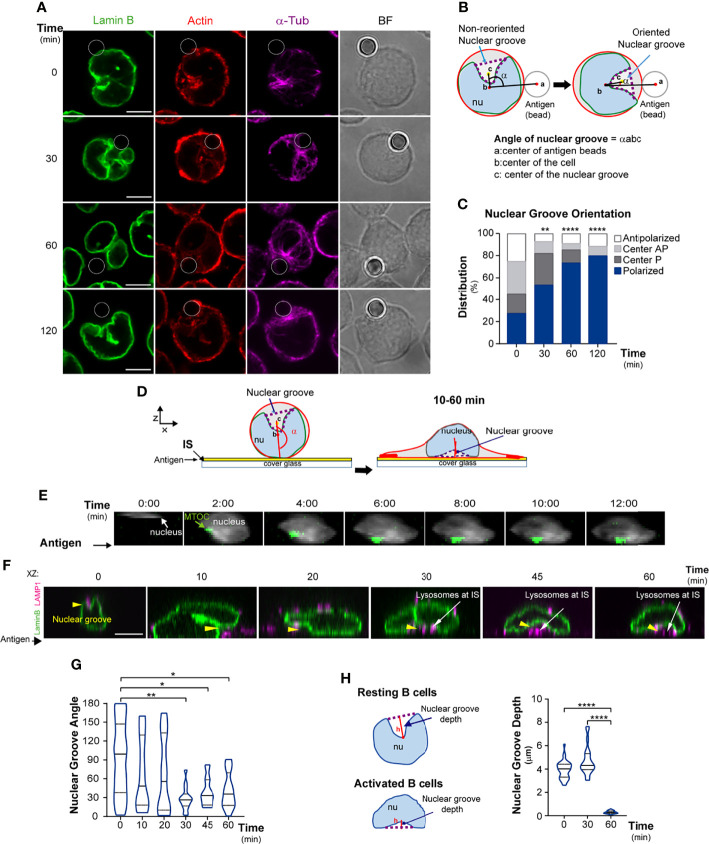
The nucleus reorients towards the B cell immune synapse. **(A–D)** Representative confocal images of B cells stained for the nucleus (Lamin B, green), actin (phalloidin, red), and microtubules (α-tubulin, magenta). Cells were incubated with antigen-coated beads (white circles) for 0, 30, 60, and 120 min. **(B)** Scheme depicting method used to measure the orientation of the principal nuclear groove. **(C)** Percentage of cells with polarized, central, or non-polarized nuclei with respect to the antigen; n≥90. **(D)** Scheme of nuclear groove rotation in B cells seeded on antigen-coated dishes. **(E)** Visualization of nucleus rotation (Hoechst, blue) and MTOC (centrin-GFP) in B cells seeded on antigen-coated dishes (filmed every 2 min). **(F, G)** Representative confocal images and quantification of nuclear groove reorientation in B cells seeded on antigen-coated dishes for indicated times, orientation is indicated between 0-180°. Nuclei (Lamin B): green; lysosomes (LAMP1): magenta; yellow arrowheads: nuclear groove; n≥52 cells. **(H)** Scheme and quantification of the nuclear groove depth, measuring the height or distance between the point of the nuclear groove farthest from a line traced between the two nuclear lobe apices. All scale bars are 5 µm. Statistical analyses: Kruskal-Wallis with Dunn’s multiple comparisons tests **(C, H)** and unpaired t-tests **(G)**. *p < 0.05, **p < 0.01, ****p < 0.0001.

In B cells activated with antigen-coated beads, reorientation of the nucleus was calculated as follows: A vector was projected between the centers of mass of the bead (a) and cell (b); and a second vector between the centers of mass of the cell and nuclear groove (c) which was manually delineating based on the staining with lamin B (**Figure 1B**). The angle between these vectors was used as a measurement of reorientation. The percentage of cells with the nuclear groove oriented toward the bead (Polarized) were defined as those with angles between 0° and 45°; a centered nuclear groove was associated to angles between 45° and 135° (Centr AP) and antipolarized nuclei were associated to angles between 135° and 180°. In the case of B cells activated on antigen-coated dishes, reorientation of the nucleus was calculated by measuring the angle between the vector projected between the centers of mass of main groove and the cell (c) and the vector formed between the cell center of mass and the synaptic plane (**Figure 1D**).

#### Measurement of the Nuclear Groove Depth

For each cell, the height (depth) of the nuclear groove was measured using Hoechst-stained images, which were segmented into stacks using a custom FIJI script with user-defined points and line segments. The height (h) of the nuclear groove was measured as the distance between the point of the nuclear groove farthest from a line traced between the two nuclear lobe apices. Values were normalized with respect to the cell diameter which was estimated with a straight line segment drawn across the cell.

#### MTOC and LAMP Polarity Indexes

These were determined as previously described (37). Briefly, we manually selected the location of the MTOC, delimited the cell and obtained the center of mass of the cell, (Cellmc) and the bead (Beadmc). The position of the MTOC was projected on the vector defined by Cellmc and Beadmc axis (Pj-mtoc). The MTOC polarity index was calculated by dividing the distance between Cellmc and Pmtoc and the distance between Cellmc and Beadmc. The index ranged from -1 (anti-polarized) to +1 (fully polarized). LAMP polarity index was calculated using the center of mass of the LAMP1 staining and proceeding as described for MTOC polarity index. Lysosomes at the Immune synapse in the bead model (lysosome rings), were quantified by measuring the LAMP1 fluorescence intensity in the circular area closely surrounding the bead (3.5 μm) and normalized by total LAMP1 fluorescence intensity.

#### Measurement of Nuclear or Actin Volume

Confocal image stacks were first filtered by FIJI using the Trainable Weka Segmentation plugin (38), to enhance membrane signals. Next, 2D threshold filters were applied within SCIAN-Soft in order to produce binary images of the nuclear/actin fluorescent signal as regions of interest (ROIs). *Actin surrounding the nucleus* was measured by defining nuclear and actin ROIs using a logical filter to obtain the intersection between the actin ROIs and the nucleus ROI. In each case (nucleus, actin, and nuclear-actin), adjacent ROIs along the z axis were connected to define 3D ROIs, and their volume was quantified by voxel counting.

#### Measurement of Nuclear Groove Area in Primary B Cells

Nuclear groove area was calculated in primary B cells by manually delineating the principal groove at its maximum size (from staining with Lamin B).

#### Overlapping Fraction of Actin With Nuclear Lamin B

2-dimensional intersections were computed with a FIJI script for all z-slices with the following steps: i) segmented images of Lamin B were obtained with a random forest classification model, trained within FIJI with the Weka plug-in [Ref. Weka (36)], and segmented Lamin B images were eroded in 1 pixel to refine boundaries; ii) intersection of actin and Lamin B segmented signals was computed, a 2-pixel distance tolerance (~146 nm) was set to account for the possible light diffraction effects in the microscope setup, and a dilation of 2 pixels was applied to the actin channel; iii) finally, pixel counting was used to quantify the ratio of intersection/total signal size (the volume of Lamin B-actin intersection divided by total volume of Lamin B).

#### Measurement of Complete Nuclear Groove Rotation

B cells were activated on antigen-coated dishes, then images were resliced (FIJI program) and the rotation of the nucleus was measured by taking the angle resulting from the baseline between the two main lobes and the immune synapse plane (**Figure 3D**). This analysis was performed using the FIJI program Analyze angles.

#### Radial Distribution of Lysosomes or the BCR Within Z Sections

values were calculated by measuring their mean fluorescence intensity (MFI) in the cell at each z-slice (0.2 µm) from the Immune synapse (defined by the contact area on antigen coated dishes) for a total of 10 z-slices. For normalization, MFI values were divided by the total fluorescence and multiplied by 100.

#### Segmentation and Position of Lysosome Clusters Relative to the Nucleus at the Synaptic Plane

Using a custom-made FIJI macro, the position of largest LAMP1^+^ lysosome cluster was calculated with respect to the nucleus (stained with Hoechst), where two sections were defined: 1) inside the perinuclear region and 2) outside of the perinuclear region. Lysosome clusters partially positioned within perinuclear region were considering as being inside.

#### Distribution of Exo70

The xy radial distribution of exo70 in resting and activated B cells was calculated with the “Radial Profile” FIJI plugin available at https://imagej.nih.gov/ij/plugins/radial-profile.html. By setting a reference point for the MTOC, the MFI was calculated and plotted for circular regions for a growing radius, from 0 to 2 µm.

### Statistical Analysis

Statistical analysis was performed with GraphPad Prism v9 (GraphPad Software; San Diego, CA). Shapiro-Wilk test was used to contrast the normality of the data set and the analytical test was used accordingly (parametric or nonparametric). P values were calculated using different tests, as indicated in figure legends.

## Results

### B Cells Change Their Nuclear Morphology and Reorient Their Nuclear Groove Toward the Immune Synapse

We first evaluated whether B cells change their nuclear morphology upon activation by immobilized antigens. To this end, we labeled the nuclear lamina of B cells under resting or activation conditions, using anti-Lamin-B, which detects only the Lamin expressed in these cells (39). We also labeled microtubules (α-tubulin) and actin (phalloidin) and performed 2D and 3D reconstructions (**Figures S1A, B**). Resting B cells displayed a lobular nucleus (3-4 lobes). The MTOC was closely associated with the nucleus, inside the main nuclear groove or within a principal intra-lobular space, similar to recent studies in hematopoietic cells (40) (**Figure S1A**). In resting cells, the nucleus occupied 70% of total cell volume (**Figure S1C**). We next evaluated nuclear morphology under activation conditions by incubating B cells with 3-μm beads coated with antigen, mimicking formation of an immune synapse (13). Upon activation (30-60 min), B cells re-positioned their nuclear groove towards the antigen (**Figures 1A–C**), and this process was coupled to MTOC polarization (**Figures S1D, E**). At later time points, we found that the antigen-coated bead became accommodated within the main nuclear groove near the MTOC, suggesting that B cells adapt their nuclear position and morphology to create a space to bring the MTOC closer to the synaptic interface. To confirm that this process was associated to BCR engagement, we examined the effect of WGA-coated beads, an irrelevant ligand that interacts with cell surface glycans. Indeed, B cells interacting with WGA-coated beads did not trigger reorientation of the nuclear groove or repositioning of the MTOC toward the synapse (**Figure S1F**).

To further characterize changes in nuclear shape following nuclear reorientation, we used another experimental setup where the antigen was fixed to coverslips, which allowed us to evaluate nuclear movement or rotation toward the antigen contact site (**Figure 1D**). To monitor dynamic changes in nuclear morphology and MTOC polarization simultaneously during B cell activation, we performed live imaging of centrin-GFP-expressing B cells labeled with Hoechst and filmed the cells every 2 min using epifluorescence microscopy. We observed that, as the MTOC became positioned towards the synaptic membrane, the nucleus rotated and changed its morphology (**Figure 1E**). Considering that MTOC polarization to the immune synapse guides the recruitment of lysosomes to this region for antigen extraction, we characterized changes in nuclear morphology together with lysosome positioning. B cells were seeded onto antigen-coated dishes for various time points, and we fixed and labeled for nucleus (Lamin B) and lysosomes (LAMP1). Notably, we observed that after 30 min, the nuclear groove was fully reoriented towards the central region of the immune synapse (**Figures 1F, G**), where lysosomes progressively accumulated. We further characterized changes in nuclear morphology, quantifying the depth (height) of the nuclear groove, defined as the distance between the point of the nuclear groove farthest from a line traced between the two nuclear lobe apices. Our analysis shows that B cells activated on antigen-coated surfaces decreased the depth of their nuclear groove facing the immune synapse (**Figure 1H**) revealing that this perinuclear region becomes closely located to the synaptic membrane. Interestingly, B cells interacting with multiple antigen-coated beads displayed larger nuclear grooves, suggesting that they open the groove according to amount of immobilized antigen (**Figure S1G**). These results reveal that the nuclear morphology of B cells changes throughout the various stages of activation and shows that lysosomes are accommodated in the central region between the nucleus and synaptic membrane inside the nuclear groove. Thus, B cell engagement with immobilized antigens triggers changes in nuclear morphology and reorientation of the nuclear groove toward the center of the immune synapse.

### Cytoskeleton-Dependent Nuclear Reorientation to the B Cell Immune Synapse

**Figure 2:**
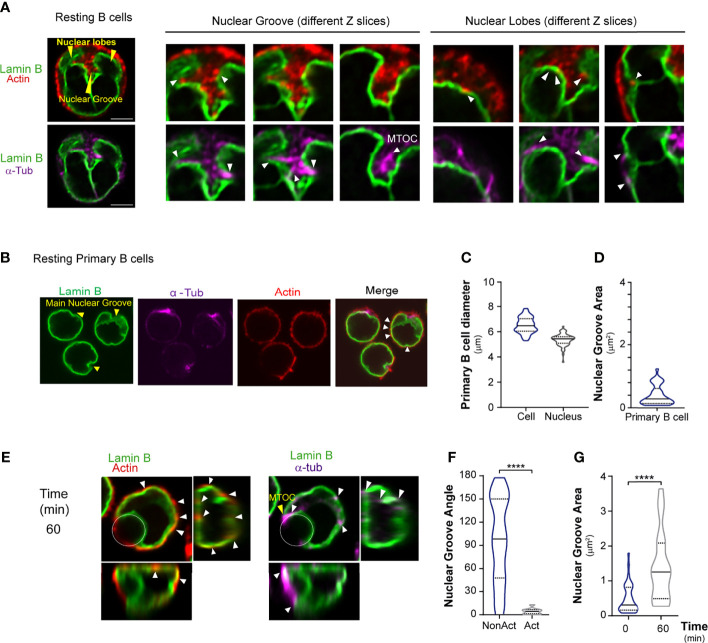
Nuclear shape is regulated by cytoskeleton stability. **(A)** Confocal images showing actin cytoskeleton (phalloidin, red) and microtubules (α-tubulin, magenta) strongly associated with the nucleus (Lamin B, green) in resting B cells. Arrowheads: nuclear groove; lobes: yellow; connection between cytoskeleton and nucleus: white. Scale bar 5 µm. **(B)** Representative confocal images of resting mouse primary B cells; stain colors/arrowheads as indicated as in **(A)**. Primary B cells with a nucleus that occupies almost the entire area of the cell. **(C)** Diameter of primary B cells and their nuclei and **(D)** nuclear groove area. **(E)** Representative confocal images of primary B cells incubated with antigen-coated beads for 60 min. White circles indicate bead position. Arrowheads: nuclear groove. **(F)** Nuclear orientation, measured as the angle of the nuclear groove. n>55, unpaired t-tests; ****p < 0.0001. **(G)** Nuclear groove area upon activation. n>55, Mann-Whitney test; ****p < 0.0001; from three independent experiments.

Nuclear morphology is known to be controlled by both actin and microtubules (41–46). We verified association of these structures with the B cell nucleus. To characterize the connection between actin and nucleus, resting or activated B cells were labeled with Lamin B, actin (phalloidin), and microtubules (α-tubulin). We observed that under resting conditions, the actin cytoskeleton and microtubules were strongly associated with the nucleus. Inside the main nuclear groove, nascent microtubules emerging from the MTOC closely surrounded each nuclear lobe (**Figure 2A**). We could also visualize the actin pool surrounding the MTOC, which was enriched inside the nuclear groove. Interestingly, actin and microtubules were associated with nuclear groove borders and were enriched in lobe vertices and curvatures as well as on top of the nucleus. These structures, therefore, may be involved in regulating nuclear positioning. Importantly, studies in primary B cells with cellular and nuclear diameters of 6.5 μm and 5.36 μm, respectively (**Figures 2B, C**), revealed smaller nuclear grooves (0.4 μm^2^ area) (**Figure 2D**) and behavior similar to that of the B cell line. These cells also reoriented their nuclear groove (**Figures 2E, F**) and adjusted the size of the groove according to the amount of antigen present (**Figures 2E–G**).

We further assessed the role of the cytoskeleton in nuclear morphology by evaluating the effects of drugs that perturb microtubules (Nocodazole, Taxol) or actin (Latrunculin A). Nuclear morphology was evaluated in resting B cells incubated with each drug for 30 min and labeled for Lamin B. 3D reconstructions of these cells revealed that all drugs decreased the total nuclear volume (**Figure S2A**), indicating that nuclear morphology in B cells is tightly coupled to cytoskeleton networks, as observed in other cell types. We next analyzed nuclear rotation and MTOC polarization in the presence of these drugs under activating conditions and also evaluated the effect of SAHA (increases microtubules acetylation), Blebbistatin (myosin II inhibitor), and Cytochalasin D, another actin depolymerizing drug. Drugs were added 10 min after activation. Our results show that the orientation of the nuclear groove and the MTOC towards the immune synapse were significantly impaired when microtubules or actin cytoskeleton were perturbed (**Figures S2B–D**). These results confirm a strong association between nuclear groove reorientation and MTOC positioning upon antigen activation. Importantly, no effects on MTOC and nucleus reorientation were observed upon treatment with Blebbistatin, indicating that these processes did not rely on myosin II activity.

Given that actin and microtubules regulate nuclear morphology and orientation in B cells, we next studied associations between cytoskeleton and the nucleus asked whether they were regulated during activation. To this end, we assessed changes in the composition of perinuclear-associated proteins in resting and activated B cells by isolating and examining the composition of nuclear, perinuclear, and cytoplasmic fractions from resting and activated B cells (**Figures S3A, B**). We focused on the perinuclear fraction, which contains proteins bound directly or indirectly to the inner or outer nuclear membrane (34). Analysis and quantification of cytoskeletal components in perinuclear fractions revealed that actin decreased after 30 min of the activation and later recovered to original levels (**Figures S3C, D**). This result is consistent with a previous report showing that B cells deplete actin from the perinuclear region in order to promote MTOC repositioning to the immune synapse (17, 37). Perinuclear α-tubulin and γ-tubulin levels decreased markedly upon activation. This effect dissipated after 30 and 60 min of activation, and original levels were restored. These results suggest that, in resting B cells, microtubules are associated with the nucleus but become less coupled to this organelle as it rotates upon activation. In terms of the nucleoskeleton, Lamin B levels in the perinuclear fraction gradually decreased during activation, suggesting that Lamin B became less coupled to the nuclear membrane. Moreover, we found that nuclear membrane-associated proteins Nesprin-1, Sun-1, Sun-2 (46) levels in perinuclear fractions did not change over the activation time course (**Figures S3C–E**), indicating that their associations with the nucleus were stable, unlike those of actin, microtubules, and Lamin B, which showed dynamic phases of nuclear association and disassociations. Overall, our results show that the association between actin and microtubules with the nuclear envelope change upon B cell activation, promoting nuclear reorientation toward the immune synapse.

### B Cells Depend on Conserved Nuclear-Cytoskeleton Proteins, Nesprin-1, and Sun-1 to Regulate Nuclear Morphology

**Figure 3:**
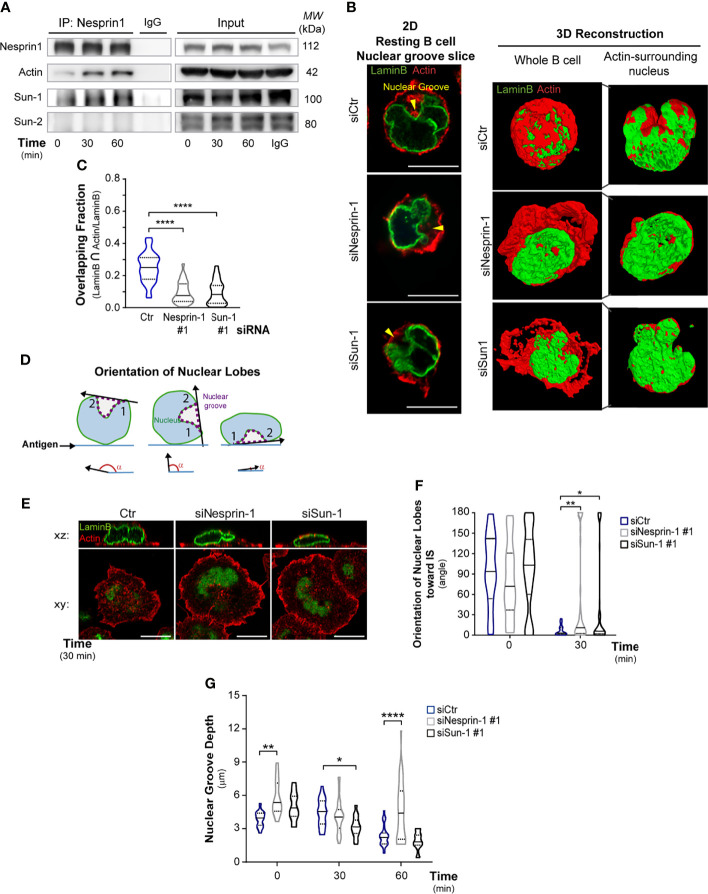
Nesprin-1 and Sun-1 regulate nuclear shape during B cell activation. **(A)** Nesprin-1 immunoprecipitation (IP) assay to detect LINC complex (Nesprin-Sun) formation in resting or activated B cells for indicated times; data represent three independent experiments. **(B)** Left: Representative confocal images of nuclear groove slice in control, Nesprin-1-, and Sun-1-silenced B cells under resting conditions. Scale bar: 10 μm. Right: 3D reconstruction images showing whole B cells (total volume of actin and Lamin B); and logical filter applied on actin signal, showing only actin surrounding the nucleus. **(C)** 3D measurement of the intersections between actin and Lamin B by segmented signals. Quantification of the ratio of intersection of Lamin B and the actin signal divided by the total lamin B signal; n≥48. **(D-F)** Measurement of nuclear groove rotation in Nesprin-1-, and Sun-1-deficient B cells. Controls and silenced cells were incubated on antigen-coated dishes for indicated times. Scheme depicting the method used to measure nuclear orientation towards the synaptic plane **(D)**, representative confocal images **(E)**, and quantification of complete nuclear lobes rotation **(F)** between 180° – 0°; n≥60 cells from two independent experiments. **(G)** Quantification of the nuclear groove depth (height) in control, Nesprin-1-, and Sun-1-silenced B cells under resting and activating conditions. n≥55. Lamin B: green; actin: red in all images. Statistical analyses: Kruskal-Wallis with Dunn’s multiple comparisons tests **(C)** and two-way ANOVA with Sidak’s multiple comparison tests **(F, G)**; *p < 0.05, **p < 0.01, and ****p < 0.0001.

Nesprin and Sun proteins regulate nuclear shape and positioning in various cell types and frequently form complexes in order to perform these effector functions (18, 47, 48). We thus evaluated whether such complexes could be formed during B cell activation. For this purpose, we performed immuno-precipitation assays with nuclear membrane proteins, which revealed that, under resting conditions, Nesprin-1 formed a complex with Sun-1 but not with Sun-2. Upon activation, Nesprin-1 and Sun-1 continued to interact and Nesprin-1 becoming associated with actin (**Figure 3A**). These results indicate that complexes formed by nuclear envelope proteins, Nesprin-1, and Sun-1 interact with actin and may regulate nuclear morphology in B cells, as observed in other cell types. To evaluate their role in regulating nuclear morphology during B cell activation, we silenced Nesprin-1 or Sun-1, achieving a 90% and 80% decrease in their expression levels, respectively (**Figure S4A**). Next, we performed 2D and 3D analyses of confocal images of resting B cells stained for the nucleus (Lamin B) and actin (**Figure 3B**). These analyses, determined by the intersection of actin and Lamin B segmented signals, revealed that the cortical actin cytoskeleton (red) of control cells covered the nucleus (green), forming a sphere, with 25% of the nucleus overlapping with perinuclear actin (**Figure 3C**). In contrast, Nesprin-1- or Sun-1-silenced B cells lost the spherical form of the cortical actin cytoskeleton and displayed ruffles that extended away from the nucleus. The proportion of perinuclear actin associated with the nucleus decreased to 8%, suggesting that Nesprin-1 and Sun-1 facilitate the connection between actin and nucleus in B cells (**Figures 3B, C**). Next, we evaluated whether Nesprin-1 and Sun-1 regulate nuclear rotation. To this end, B cells were seeded onto antigen-coated dishes, fixed and stained for lamin B and actin. Nuclear orientation was quantified by measuring the angle formed between the line traced between the two nuclear apices of the lobules and the synaptic plane, as indicated in the scheme shown in **Figure 3D**. This analysis revealed that Sun-1- and Nesprin-1-silenced B cells were unable to completely reorientate their nuclear groove to the immune synapse, forming larger angles between their nuclear lobules and the synaptic plane (**Figures 3E, F**).

Additionally, we evaluated the role of Nesprin-1 and Sun-1 in regulating nuclear morphology, by measuring the nuclear groove depth in these cells under resting and activating conditions. As described above, upon activation with immobilized antigens, control cells decrease the depth of their nuclear groove facing the immune synapse. In contrast, Nesprin-1-silenced B cells the depth of their nuclear groove was higher in resting conditions, compared to control cells and did not change upon activation (**Figure 3G**). Taken together, these results show that Sun-1 and Nesprin-1 regulate nuclear morphology in resting B cells and upon interaction with immobilized antigens.

### Nesprin-1 and Sun-1 Are Required for B Cells to Establish an Organized Immune Synapse

**Figure 4:**
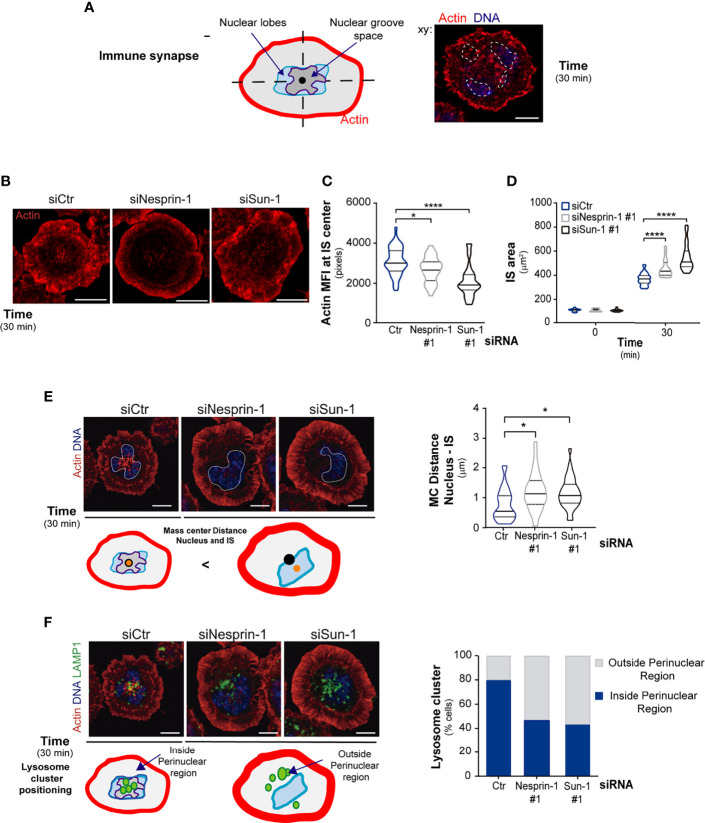
Nesprin-1 and Sun-1 regulate immune synapse organization. **(A)** Scheme depicting a mature immune synapse formed by B cells. Arrows indicate nuclear groove space and position of nuclear lobes at the center of the immune synapse. Right side: a confocal image of a B cell seeded on an antigen-coated dish for 30 min, fixed and stained for the nucleus (Hoechst, blue) and actin (phalloidin, red). Nuclear lobules are highlighted with a white line. **(B–D)** Representative confocal images of the actin signal at the immune synapse of B cells silenced for Nesprin-1 or Sun-1, labeled and activated as in A; quantification of central actin MFI **(C)** and peripheral actin, which was used to measure immune synapse area, n≥60 **(D)**. **(E, F)** Confocal images of B cells activated as in A and schemes showing nuclear and lysosome positioning at synaptic center. **(E)** Upper panel shows cells stained for actin (red) and nucleus (Hoechst in blue, delineated with a white segmented line). Schemes below indicate, the center of mass of the immune synapse (black dot) and the nucleus center of mass (orange dot). Quantification of distance between the nucleus and immune synapse mass center (MC); n>60. **(F)** Upper panel shows cells stained as in E and stained for lysosomes (LAMP1, green). Schemes below indicate, lysosome positioning respect to the nucleus and the immune synapse. Quantification of lysosome cluster located outside or inside of the perinuclear region, n>60. All scale bars 5 µm. Statistical analyses: Kruskal-Wallis with Dunn’s multiple comparisons tests; *p < 0.05, ****p < 0.0001.

Given that Nesprin-1 and Sun-1 are required to maintain the connection between actin and nucleus in resting cells, we asked whether they also regulate actin reorganization at the immune synapse, where the nucleus becomes tightly repositioned. To this end, we measured actin levels at the center of the immune synapse where the nuclear groove sits. We also measured peripheral actin, which limits the area of the synaptic membrane (**Figure 4A**). Our results show that Nesprin-1- and Sun-1-silenced B cells display significant defects in actin reorganization at the immune synapse. Such defects include decreased actin levels at the center of the synapse (**Figures 4B, C**) and the formation of large peripheral lamellipodia, resulting in increased spreading areas compared to control cells (**Figure 4D**). Taken together, these results show that Sun-1 and Nesprin-1 regulate actin organization surrounding the nuclear as well as at the immune synapse.

Having shown that Nesprin-1 and Sun-1 are required to maintain connection between actin and nucleus and regulate actin at the immune synapse, we next evaluated their role in synapse organization. To this end, control or Sun-1/Nesprin-1-silenced B cells were activated on antigen-coated dishes. The distribution of the BCR, lysosomes, and nucleus was evaluated in terms of their localization with respect to the immune synapse (z sections) as well their position within this region (central or peripheral). Regarding the BCR, control cells almost completely internalized the BCR from the cell surface after 15 min of ligand stimulation, revealed by staining of the receptor in non-permeabilized conditions. In contrast, Nesprin-1- and Sun-1-silenced cells displayed approximately 50% of this receptor at the cell surface after ligand stimulation, suggesting that they present defects in BCR internalization (**Figures S5A–C**).

Analysis of the nucleus revealed that control cells positioned this organelle at the center of the immune synapse, whereas in Nesprin-1 and Sun-1-silenced cells it was displaced from the synaptic center (**Figure 4E**). Similar defects were observed in lysosome positioning, where lysosomes were polarized to the immune synapse in both cell types (**Figure S5D**) but did not accumulate within the perinuclear space at the center of the immune synapse in Nesprin-1 and Sun-1-silenced (**Figure 4F**). These observations suggest that correct nuclear positioning is closely coupled to the proper localization of lysosomes.

Overall, silencing nuclear envelope proteins alters the distribution of actin, BCR, and the MTOC as well as lysosome recruitment towards the center of the immune synapse, highlighting a functional link between the regulation of nuclear morphology and immune synapse organization.

### Nesprin-1 and Sun-1 Facilitate Antigen Extraction by Regulating Exo70 Positioning at the Immune Synapse

**Figure 5:**
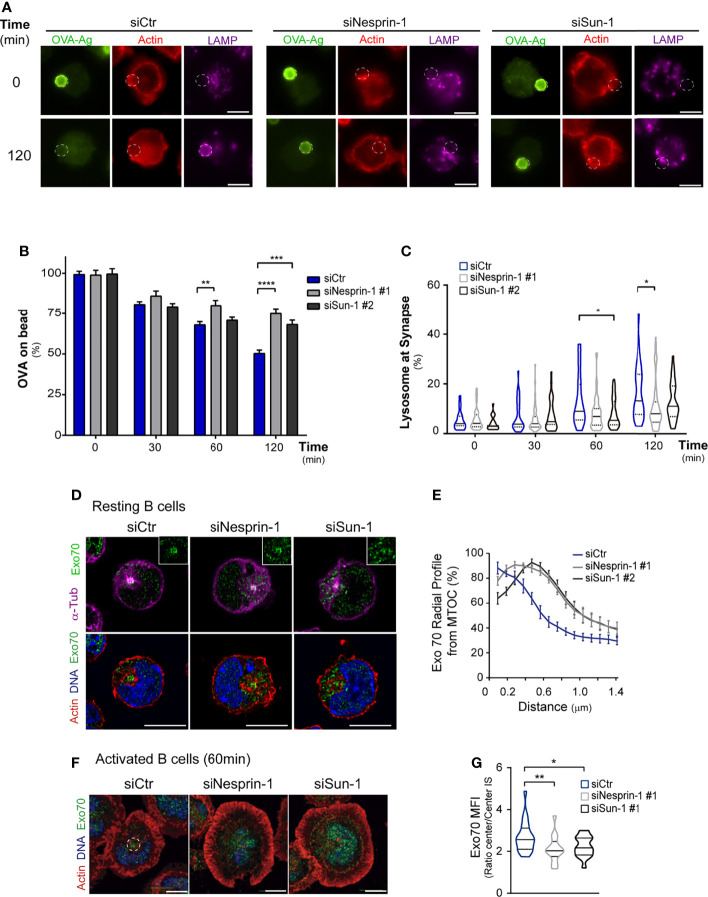
Antigen extraction relies on Nesprin-1 and Sun-1. **(A)** Epifluorescence images of control or Nesprin-1- and Sun-1-silenced B cells incubated with antigen-coated beads for 0 or 120 min. Cells were stained against OVA (Green), actin (red), and LAMP1 (magenta). White circles indicate bead position. Scale bar 5 μm. **(B)** Percentage of OVA remaining on antigen-coated beads; n=80, **p = 0.001; ***p = 0.0006; ****p < 0.0001. Means with SEM lines shown. **(C)** Measurement of LAMP1+ rings surrounding antigen-coated beads; n=80; *p=0.05. **(D, E)** Confocal images of silenced B cells (as in A) under resting conditions, showing Exo70 (green) with: upper panel, microtubules/MTOC (α-Tub, magenta); and lower panel, actin (red) and nucleus (Hoechst, blue). Quantification of Exo70 distribution (radial profile) from the MTOC. n>30. **(F, G)** Confocal images of silenced B cells (as in **A**) activated on antigen-coated dishes for 30 min. Exo70 (green), actin (red) and nucleus (Hoechst, blue). Quantification of Exo70 concentration in central region of immune synapse; n=40, *p < 0.05, **p < 0.01. Statistical analyses: Kruskal-Wallis with Dunn’s multiple comparisons tests **(B, C, G)**, mixed-effects analyses, and Dunnett’s multiple comparisons tests **(E)**.

Having shown that Nesprin-1 and Sun-1 regulate immune synapse organization, we next assessed their role in regulating antigen extraction capacity by measuring the amount of antigen (OVA) remaining on beads in contact with activated B cells. Indeed, Nesprin-1- and Sun-1-silenced B cells extracted less antigen compared to control cells (**Figures 5A, B**). To evaluate whether the cause of defective antigen extraction is a consequence of mispositioned lysosomes at the synaptic interface, we measured MTOC and lysosome polarization toward the antigen in control, Nesprin-1-, and Sun-1-silenced B activated using antigen-coated beads. Our results confirm that Nesprin-1- and Sun-1-silenced B cells did not display significant defects in MTOC (**Figures S6A, B**) or lysosome polarization (**Figures S6C, D**), suggesting that the movement of lysosomes toward the immune synapse was not affected. However, upon activation, we detected that these cells formed less lysosome LAMP1+ rings that typically surround the activating beads (**Figure 5C**). As described above, lysosome did not accumulate at the center of the immune synapse in Nesprin-1 or Sun-1-silenced B cells, together these results suggest that docking and secretion at the synaptic interface was impaired, thereby explaining their lower antigen extraction capacity.

To obtain mechanistic insights underlying the altered positioning of lysosomes at the immune synapse in Nesprin-1 or Sun-1-silenced B cells, we focused on Exo70, a subunit of the exocyst complex associated with the MTOC that is repositioned to the B cell immune synapse, where it is required for tethering of lysosomes to the center of the synaptic membrane (15, 49, 50). To evaluate whether Nesprin-1- or Sun-1-silenced B cells had defects in mobilizing Exo70 to the synaptic membrane, we evaluated its localization under resting and activation conditions in these cells. Using imaging analysis, we observed that Nesprin-1- and Sun-1-silenced B cells had lower levels of Exo70 associated with the MTOC under resting conditions (**Figures 5D, E**). Upon activation, Exo70 was distributed in a more dispersed fashion throughout the synaptic membrane compared to control cells, where it concentrated at center (**Figures 5F, G**). These results suggest that Nesprin-1 and Sun-1 regulate the association of Exo70 with the MTOC and its further localization to the center of the immune synapse. Thus, the altered distribution of Exo70 in Nesprin-1- and Sun-1-silenced cells could explain why lysosomes fail to accumulate at the center of the synapse in these cells, thereby leading to impaired antigen extraction.

Collectively, our results provide evidence that Nesprin-1 and Sun-1 regulate nuclear morphology and orientation towards the immune synapse of activated B cells. The connection between these nuclear envelope proteins, mainly though the actin cytoskeleton, is required to orchestrate BCR internalization and promote precise lysosome-Exo70 recruitment for efficient antigen extraction.

## Discussion

We here show that B cells adjust their nuclear morphology upon interaction with immobilized antigens. Such changes involve the reorientation of the nuclear groove towards the antigen contact site (immune synapse) and changes in nuclear groove size, which rely mainly on interactions between nuclear envelope proteins, Nesprin-1 and Sun-1 (part of the LINC complex), and the actin cytoskeleton. Indeed, actin remodeling around the MTOC, at the perinuclear region, has been previously described to occur in B cells, where BCR engagement triggers actin depletion from MTOC in a proteasome-dependent manner, thereby enabling MTOC repositioning from the perinuclear region to the synaptic membrane (37). In this report, we reveal another level of regulation showing that the LINC complex connects the nucleus to actin in order to orientate and regulate the size of the nuclear groove facing the antigen at the immune synapse. In migrating fibroblasts, movement of the nucleus away from the leading edge, enables MTOC reorientation to the migrating front. Nuclear movement is associated with actin retrograde flow, a process regulated by Cdc42 (51). Of note, Cdc42 is activated upon BCR antigen engagement, and regulates actomyosin contractions leading to MTOC, and lysosome polarization to the immune synapse (13). Thus, Cdc42 emerges as a key candidate that could regulate nuclear morphology in B cells and shall now be evaluated.

The main role of MTOC polarization in B cells is to drive lysosome recruitment and exocytosis at the immune synapse to facilitate efficient antigen extraction, a crucial step for their activation (13, 14). In this study, the higher levels of γ-Tubulin detected in perinuclear fractions under resting conditions and after longer activation times most likely reflect the tight association between the MTOC and nuclear membrane before antigen recognition, when the nucleus is fully reoriented to the immune synapse. We propose that this process is also regulated by nuclear orientation and morphology, where the nuclear groove becomes strategically positioned at the center of the synapse to enable accumulation of lysosomes at the synaptic membrane.

What are the molecular links between nuclear positioning and lysosome trafficking? We recently showed that Exo70, a component of the exocyst complex, associates with the MTOC and is recruited to the synaptic membrane of B cells to promote lysosome tethering (15). Interestingly, Exo70 has been described to interact with the lysosome-related organelle complex-1 (BLOC-1), which promotes Exo70 trafficking from the perinuclear region to the periphery of fibroblasts. BLOC-1 also interacts with dysbindin and pallidin, which are involved in nuclear positioning (52). Dysbindin- and pallidin-knockout mice showed aberrant nuclear positioning in kidney tubule cells, resulting in loss of cell polarization (53). In B cells, silencing Nesprin-1 and Sun-1 decreased the association of Exo70 with the MTOC, which displayed a more dispersed distribution throughout the synaptic membrane. Thus, LINC complex proteins influence the distribution of proteins involved in lysosome positioning and tethering at the plasma membrane. Whether Exo70 interacts with nuclear membrane proteins such as the LINC complex to regulate its positioning remains to be addressed.

Interestingly, Nesprin-1- and Sun-1-silenced B cells displayed higher levels of surface BCRs after early time points of activation with immobilized antigens, suggesting receptor internalization was defective in these cells. However, we cannot exclude that enhanced translocation of the BCR to the surface can explain these results. Nevertheless, under resting conditions both control and Nesprin-1-/Sun-1-silenced B cells have similar BCR levels at the cell surface, indicating that these cells do not display major differences in BCR trafficking at steady state. Additionally, considering that upon ligand engagement, the BCR is internalized to lysosome compartments and does not recycle (54), we favor the conclusion that that Nesprin-1- and Sun-1-silenced B cells display defects in BCR internalization upon engagement with immobilized antigens.

How do perturbations in nuclear envelope proteins, which result in nuclear mispositioning, affect the trafficking of a cell surface receptor? One possibility is that Nesprin-1 and Sun-1 affect cortical actin organization, as shown here, which could compromise recruitment of clathrin (55) and actin binding proteins, such as Abp1, known to regulate BCR endocytosis (56), or modulate clustering of receptors coupled to this internalization (57). The mechanism by which the nuclear envelope regulates cortical actin cytoskeleton organization and associated proteins involved in BCR trafficking remains to be addressed.

What other cues, in addition to BCR ligands, can modify nuclear morphology in B cells? Mechanical or physical forces that regulate intrinsic nuclear plasticity and morphology have been described in other cell types. We noticed that the morphology of the plasma membrane frequently followed the form of the nucleus, which was more evident in primary B cells. Thus, mechanosensing at the level of the synaptic membrane could be directly transmitted to the nucleus. Studies in fibroblasts suggest that Lamins A and C control the local mechanical stiffness within certain regions of the nuclear membrane, while Lamin B, in turn, mainly contributes to nuclear integrity (58). Most cells express both Lamin A and B, and silencing of Lamin A leads to irregular or deformed nuclei (59, 60). Interestingly, B cells only express Lamin B (39), suggesting that their nuclei could be less rigid. In fact, we observed that, depending on the area of the antigen-presenting surface, B cells expand their nuclear area, reflecting their high plasticity. This property would allow B cells to efficiently accommodate their cytoplasm to coordinate lysosome trafficking for antigen extraction and processing. In addition, nuclear plasticity could also regulate B cell motility inside the lymph node during the search for antigens. Indeed, a functional connection and coordination between the nucleus and MTOC has been revealed in dendritic cells during antigen exploration. Migrating dendritic cells position their nucleus at the leading edge, which acts as a sensor to find the path of less resistance, whereas the MTOC defines directionality (61). Whether B cells employ a similar strategy when searching for antigens remains unknown. We did not observe any significant impairment in MTOC polarization to the B cell immune synapse silenced for Nesprin-1 or Sun-1. However, given that in physiological environments, B cells are frequently activated by antigens associated with softer substrates, it would be interesting to evaluate MTOC polarization in the absence of LINC complex components under such conditions.

**Figure 6:**
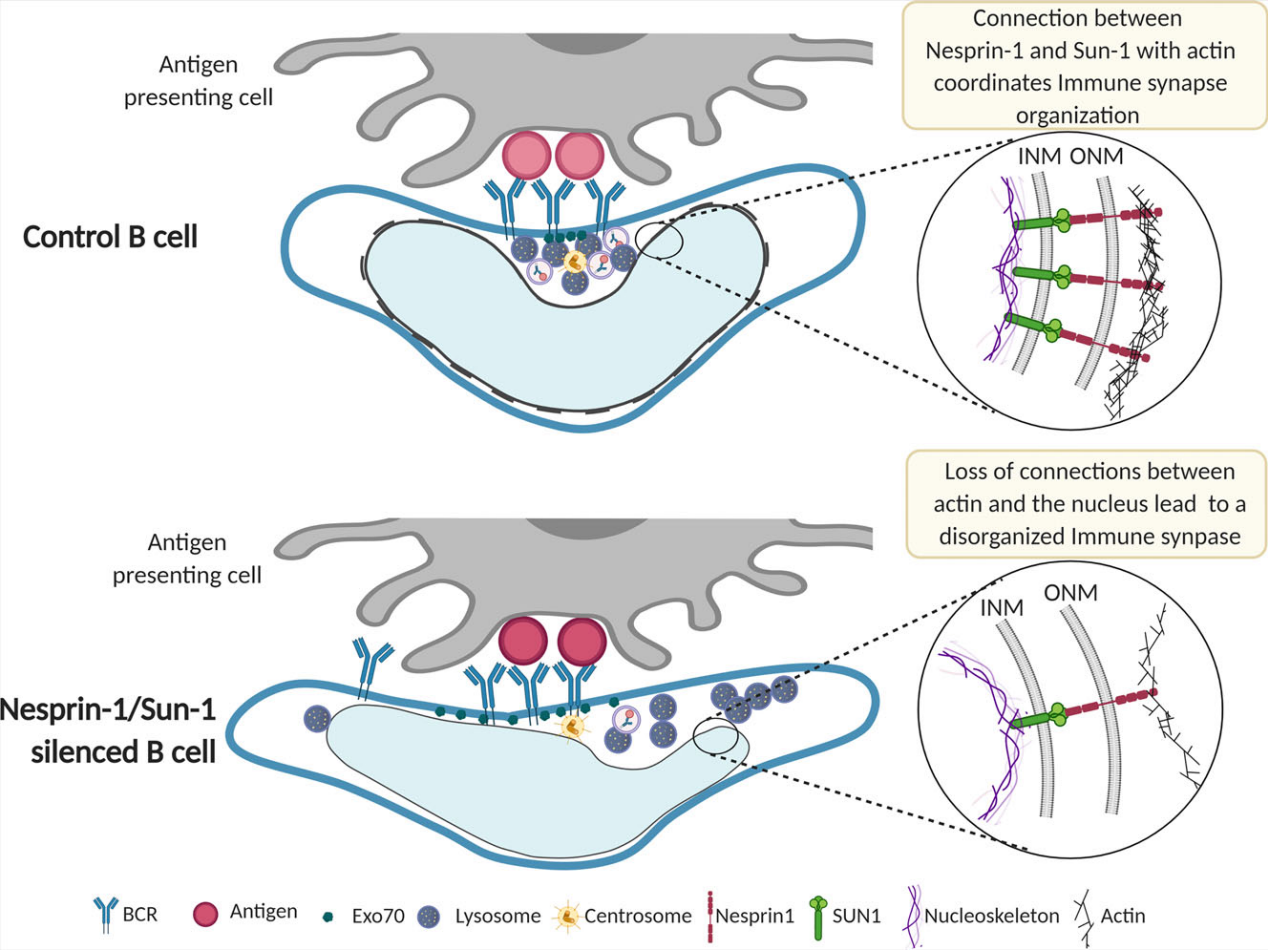
Model of immune synapse organization controlled by nuclear envelope proteins Nesprin-1 and Sun-1. Upon activation, the immune synapse formed by a B cell and antigen-presenting cell is well organized. The MTOC, Exo70, and lysosomes are recruited to the center of the immune synapse, where antigen-BCR complexes are clustered and internalized. This region is formed by the positioning of the nuclear groove, which orchestrates cytoskeleton remodeling and membrane trafficking. In Nesprin-1- and Sun-1-silenced B cells, connections between actin and the nucleus are lost; the nuclear groove fails to orient towards the antigen contact site, leading to a disorganized immune synapse. This disorganized synapse is characterized by diminished BCR clustering at the center of the synapse and defective Exo70 recruitment, impairing local tethering of lysosomes required to efficiently extract and process immobilized antigens. Further studies are required to elucidate how physical changes in the shape of the nucleus impact immune synapse organization.

Overall, this work highlights the ways in which the nucleus modifies its orientation and morphology in response to interaction with antigen-coated surfaces. Nuclear conformation adapts to the antigen contact site to define a central region, juxtaposed with the nuclear groove and limited by the nuclear lobes. In this small cell, this configuration facilitates convergence of organelles specialized in antigen extraction and processing (**Figure 6**). Thus, nuclear envelope proteins, such as Nesprin-1 and Sun-1, accommodate the intracellular architecture of B cells to control immune synapse structure and function.

## Data Availability Statement

The original contributions presented in the study are included in the article/**Supplementary Material**. Further inquiries can be directed to the corresponding authors.

## Ethics Statement

Mice protocols were approved by the Institutional Scientific Ethics Committees for Animal and Environmental Care and Research Biosafety, Pontificia Universidad Católica de Chile.

## Author Contributions

RU performed most of the experiments. OC and FC performed experiments and helped draft the manuscript. JJ-W provided tools and feedback for 2D and 3D analysis. JL helped with image analysis. JS performed co-immunoprecipitation and perinuclear fractionation assays. CR performed immunofluorescence and 3D analysis. CQ and SH provided tools for analysis and development of the project. MI-Y and JD provided tools and critical feedback on experiments and wrote the manuscript. JD also performed experiments, analysis, and conceived and supervised the project. All authors contributed to the article and approved the submitted version.

## Funding

This project was supported by research grants for FONDECYT 1171024 for JD; FONDECYT 1180900 for MI-Y; FONDECYT 1211988, DAAD 57519605, CORFO 16CTTS-66390 for SH; ICM-P09-015F for SH & JJ-W; CONICYT/ANID scholarship program for OC and JL.

## Conflict of Interest

The authors declare that the research was conducted in the absence of any commercial or financial relationships that could be construed as a potential conflict of interest.

## Publisher’s Note

All claims expressed in this article are solely those of the authors and do not necessarily represent those of their affiliated organizations, or those of the publisher, the editors and the reviewers. Any product that may be evaluated in this article, or claim that may be made by its manufacturer, is not guaranteed or endorsed by the publisher.

## Supplementary Material

Supplementary Figure 1
**(A)** Representative confocal images of a resting B cell showing various Z-slices and 3D reconstructions used to evaluate cytoskeleton-associated nuclear morphology. Cells were fixed and stained for nucleus (Lamin B, green), actin (phalloidin, red), and microtubules (α-tubulin, magenta); yellow arrowheads indicate nuclear groove. **(B)** 3D reconstructions of images, showing B cell actin cortex, nucleus, and nuclear groove, **(C)** cell, nuclear, and cytoplasmic volumes; nucleus/cell volume; and nuclear groove volume. **(D)** Centrosome polarity index with respect to immune synapse; n≥45 cells from three independent experiments. **(E)** Correlation between nuclear groove and MTOC orientation by comparison of angles with respect to immune synapse. **(F)** Representative confocal images of B cells incubated with WGA-coated beads. Quantification of nuclear reorientation and centrosome polarity toward the beads (Percentage of cells with polarized, central, or non-polarized nuclei with respect to the antigen and Centrosome polarity index with respect to immune synapse), n ≥50 **(G)** Confocal images (left) and quantification (right) of increased nuclear groove area observed in B cells interacting with multiple activating beads; stained as described in **(A)**; n=40. All scale bars 5 µm. Statistical analyses: unpaired t-tests. *p<0.05, **p<0.01, ***p<0.001, ****p<0.0001.

Supplementary Figure 2
**(A)** Resting cells were treated with cytoskeleton-disrupting drugs Nocodazole (Noco), Taxol, and Latrunculin A (LatA) for 30 min. Upper panel: 3D representative confocal images of cells stained for nucleus (Lamin B, green); lower panels: digitalization of each image and respective quantifications; n≥40. **(B)** Confocal images showing immune synapse plane of B cells incubated for 60 min with antigen-coated beads. After 10 min of activation, cells were treated with cytoskeleton-disrupting drugs as described in A or Cytochalasin D (CytD), SAHA or Blebbistatin (Bleb). White circles indicate bead position. **(C)** Percentage of cells with nuclear orientation towards immune synapse. n≥60 cells. **(D)** Percentage of cells with MTOCs oriented towards immune synapse; n≥90; three independent experiments. Statistical analyses: Kruskal-Wallis with Dunn’s multiple comparisons tests **(A, C, D)** *p<0.05, **p<0.01, ***p<0.001, ****p<0.0001.

Supplementary Figure 3 Analysis of cytoplasmic (Cyt.), perinuclear (Peri.), and nuclear (Nuc.) fractions purified from B cells. **(A, B)** Western blot of markers for each subcellular fraction: αTub, GAPDH, and actin as cytoplasmic markers; Nesprin-1 and Lamin B as perinuclear markers; and histone-3 (H3) as a nuclear marker. Resting **(A)** and activated **(B)** B cells. **(C)** Representative western blot of proteins in perinuclear fractions purified from B cells activated at different time points, as in **(B)**, did not contain histone-3 (H3). **(D, E)** Quantification of perinuclear fractions levels indicated in **(C)**. **(D)** LINC complex (Nesprin-1, Sun-1, Sun-2) and **(E)** cytoskeleton proteins (αTub, γTub, actin). Three independent experiments.

Supplementary Figure 4 Left: Nesprin-1 or Sun-1 protein and GAPDH levels from control or Nesprin-1 and Sun-1-silenced cells, detected by Western blot. Right: Nesprin-1 and Sun-1 levels normalized to GAPDH levels. n=4 independent experiments. Statistical analyses: ANOVA followed by Sidak’s multiple comparison tests. ****p<0.0001.

Supplementary Figure 5
**(A)** Confocal images of control, Nesprin-1- and Sun-1-silenced B cells activated on antigen-coated dishes for 15, 30, and 60 min. BCR (grey), actin (red), and Lamin B (green). Scale bar 5 µm. **(B)** BCR fluorescence intensity at the immune synapse (0-0.2 μm) and intracellular localization (0.4-2 μm) at each z plane. Mixed-effects analysis and Dunnett’s multiple comparisons test. n>30. * and # indicate statistical differences between control and Nesprin-1- or Sun-1-silenced cells, respectively. */# p<0.05, **/##p<0.01, ***/###p<0.001, ****/####p<0.0001. Mean and 95% CI line are shown. **(C)** Quantification of BCR remaining on the cell surface. Control and Nesprin-1- and Sun-1-silenced B cells (resting or activated for 15 min) were incubated with anti-mouse Alexa-647 to label the BCR in non-permeabilized conditions. Average levels measured from Control cells was considered as 100% for comparison rates. n≥60. **(C)** Lysosome fluorescence intensity at the immune synapse (0-0.2 μm) and intracellular localization (0.4-2 μm) according to Z distance. Cells were activated on antigen-coated dishes for 30 and 60 min. Mixed-effects analyses and Dunnett’s multiple comparisons tests confirmed no significant differences (ns).

Supplementary Figure 6
**(A, B)** Representative images of microtubules (α-tubulin, magenta) and centrosome polarity indexes for control and Nesprin-1- and Sun-1-silenced B cells, activated with antigen-coated beads at indicated times; n≥70. **(C)** Representative confocal images of silenced B cells activated as in **(A)**. Nucleus (Lamin B, green), actin (phalloidin, red), and lysosomes (LAMP1, magenta). Scale bar 5 µm. **(D)** Lysosome polarity index; n≥80. Mann-Whitney test showed no significant differences (ns) in **(B)** and **(D)**. Statistical analyses: Kruskal-Wallis with Dunn’s tests; n≥40 cells from two independent experiments. *p<0.05, **p<0.01. White circles indicate bead position.
