# Supplementary figures and images for: B Cells Adapt Their Nuclear Morphology to Organize the Immune Synapse and Facilitate Antigen Extraction
Source: Front Immunol. 2022 Feb 9;12:801164. doi: 10.3389/fimmu.2021.801164 (PMC8863768; doi:10.3389/fimmu.2021.801164)

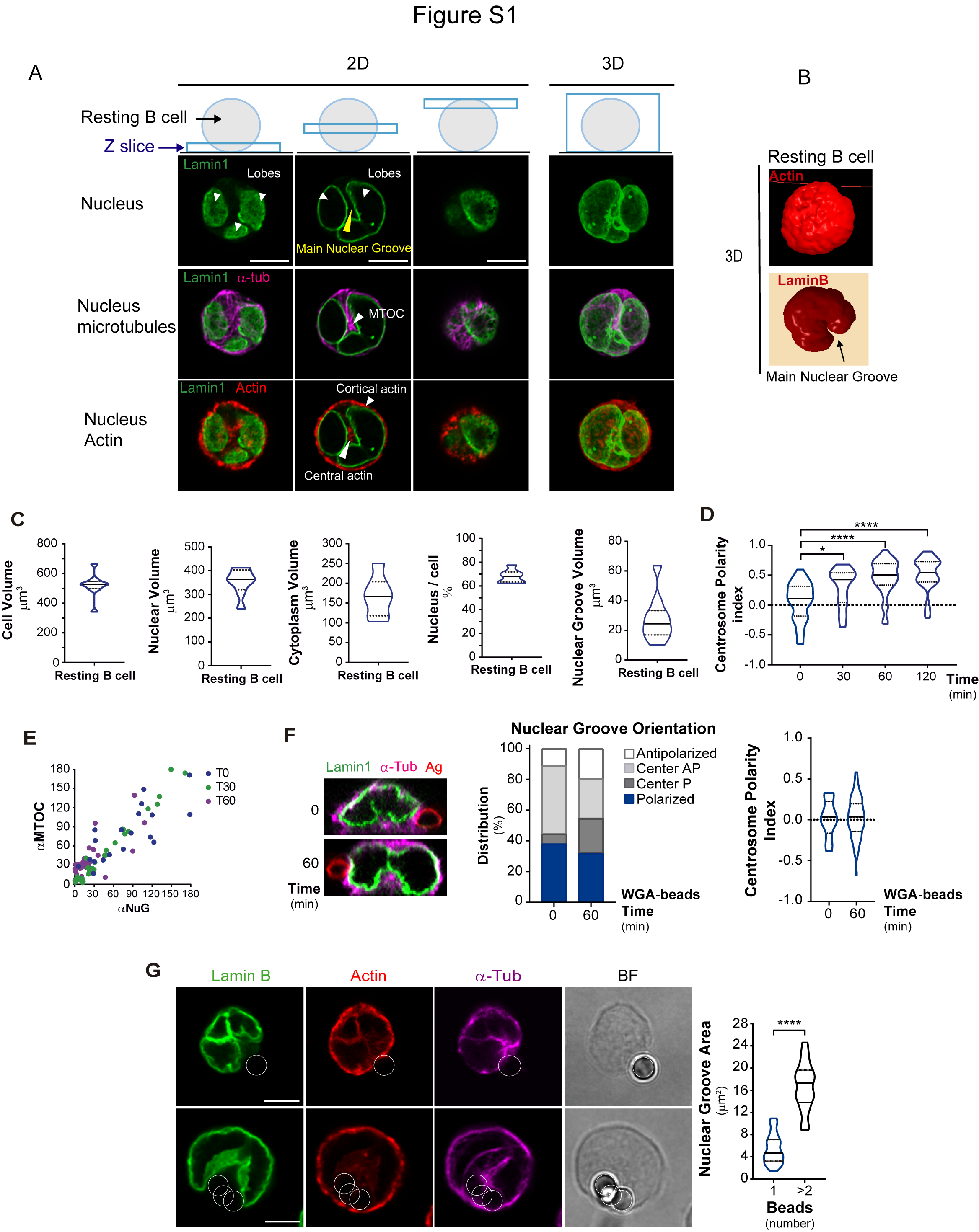

Supplement: Supplementary Figure 1 — (A) Representative confocal images of a resting B cell showing various Z-slices and 3D reconstructions used to evaluate cytoskeleton-associated nuclear morphology. Cells were fixed and stained for nucleus (Lamin B, green), actin (phalloidin, red), and microtubules (α-tubulin, magenta); yellow arrowheads indicate nuclear groove. (B) 3D reconstructions of images, showing B cell actin cortex, nucleus, and nuclear groove, (C) cell, nuclear, and cytoplasmic volumes; nucleus/cell volume; and nuclear groove volume. (D) Centrosome polarity index with respect to immune synapse; n≥45 cells from three independent experiments. (E) Correlation between nuclear groove and MTOC orientation by comparison of angles with respect to immune synapse. (F) Representative confocal images of B cells incubated with WGA-coated beads. Quantification of nuclear reorientation and centrosome polarity toward the beads (Percentage of cells with polarized, central, or non-polarized nuclei with respect to the antigen and Centrosome polarity index with respect to immune synapse), n ≥50 (G) Confocal images (left) and quantification (right) of increased nuclear groove area observed in B cells interacting with multiple activating beads; stained as described in (A); n=40. All scale bars 5 µm. Statistical analyses: unpaired t-tests. *p<0.05, **p<0.01, ***p<0.001, ****p<0.0001. [file Image_1.tif]

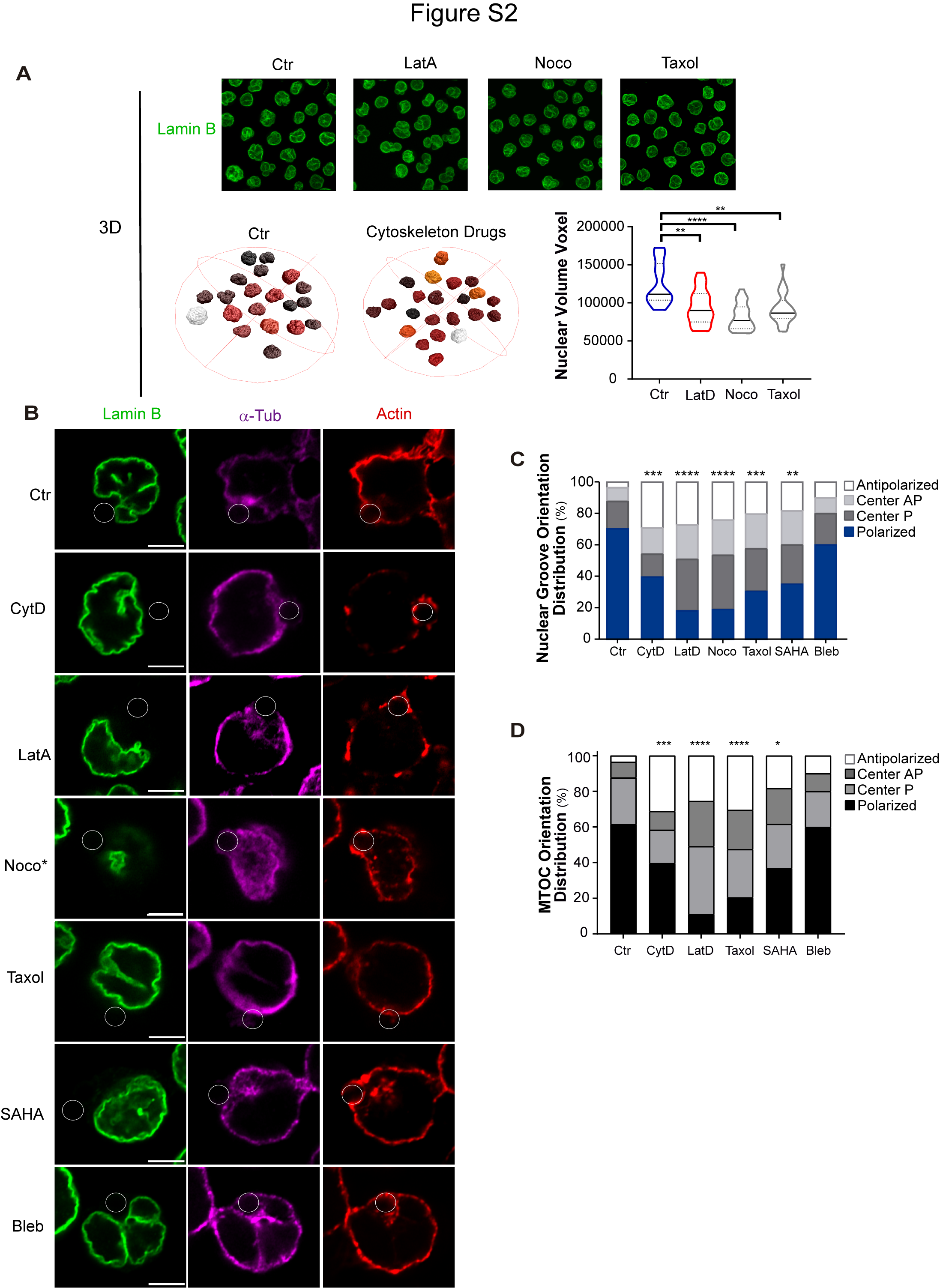

Supplement: Supplementary Figure 2 — (A) Resting cells were treated with cytoskeleton-disrupting drugs Nocodazole (Noco), Taxol, and Latrunculin A (LatA) for 30 min. Upper panel: 3D representative confocal images of cells stained for nucleus (Lamin B, green); lower panels: digitalization of each image and respective quantifications; n≥40. (B) Confocal images showing immune synapse plane of B cells incubated for 60 min with antigen-coated beads. After 10 min of activation, cells were treated with cytoskeleton-disrupting drugs as described in A or Cytochalasin D (CytD), SAHA or Blebbistatin (Bleb). White circles indicate bead position. (C) Percentage of cells with nuclear orientation towards immune synapse. n≥60 cells. (D) Percentage of cells with MTOCs oriented towards immune synapse; n≥90; three independent experiments. Statistical analyses: Kruskal-Wallis with Dunn’s multiple comparisons tests (A, C, D) *p<0.05, **p<0.01, ***p<0.001, ****p<0.0001. [file Image_2.tif]

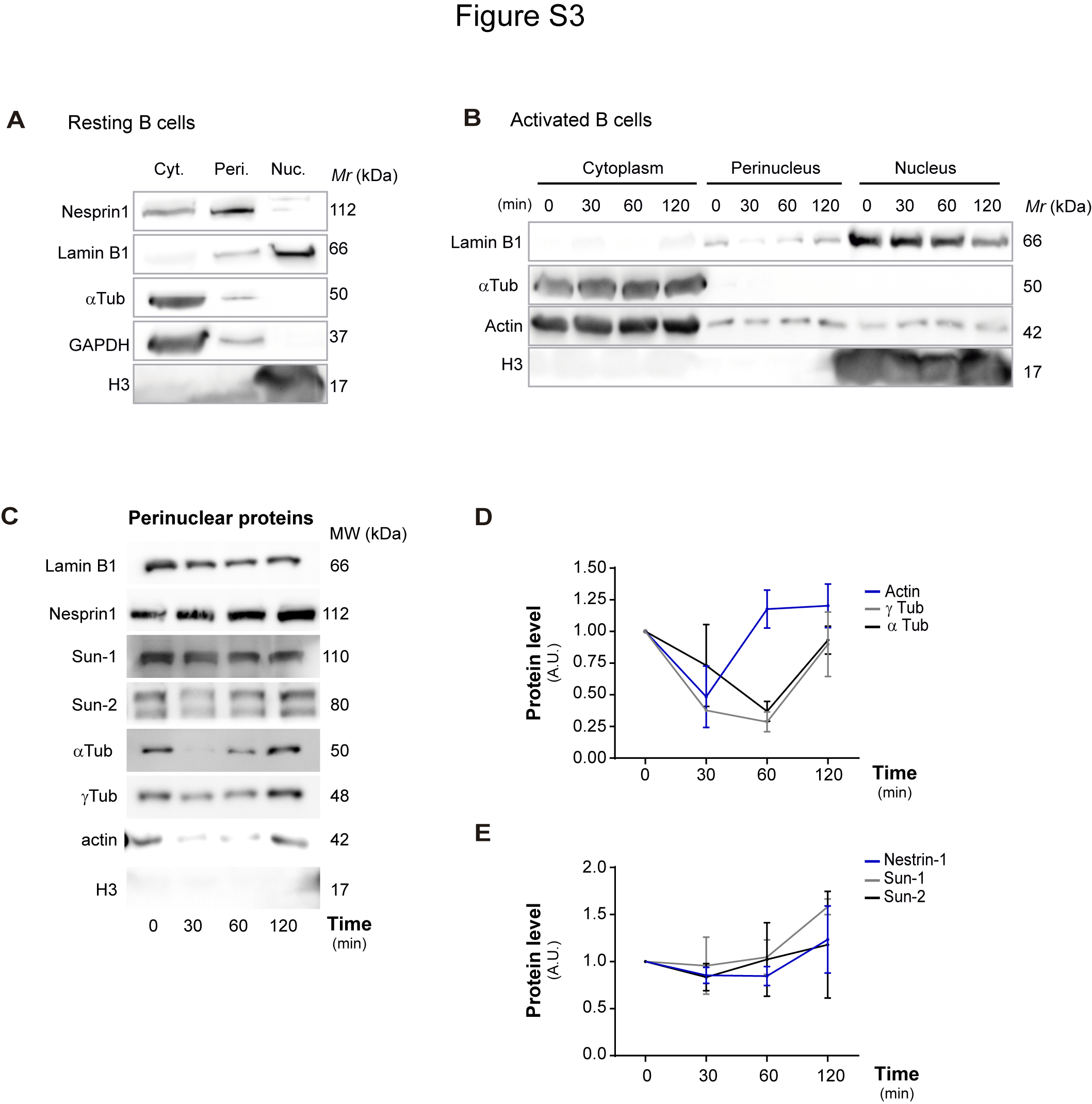

Supplement: Supplementary Figure 3 — Analysis of cytoplasmic (Cyt.), perinuclear (Peri.), and nuclear (Nuc.) fractions purified from B cells. (A, B) Western blot of markers for each subcellular fraction: αTub, GAPDH, and actin as cytoplasmic markers; Nesprin-1 and Lamin B as perinuclear markers; and histone-3 (H3) as a nuclear marker. Resting (A) and activated (B) B cells. (C) Representative western blot of proteins in perinuclear fractions purified from B cells activated at different time points, as in (B), did not contain histone-3 (H3). (D, E) Quantification of perinuclear fractions levels indicated in (C). (D) LINC complex (Nesprin-1, Sun-1, Sun-2) and (E) cytoskeleton proteins (αTub, γTub, actin). Three independent experiments. [file Image_3.tif]

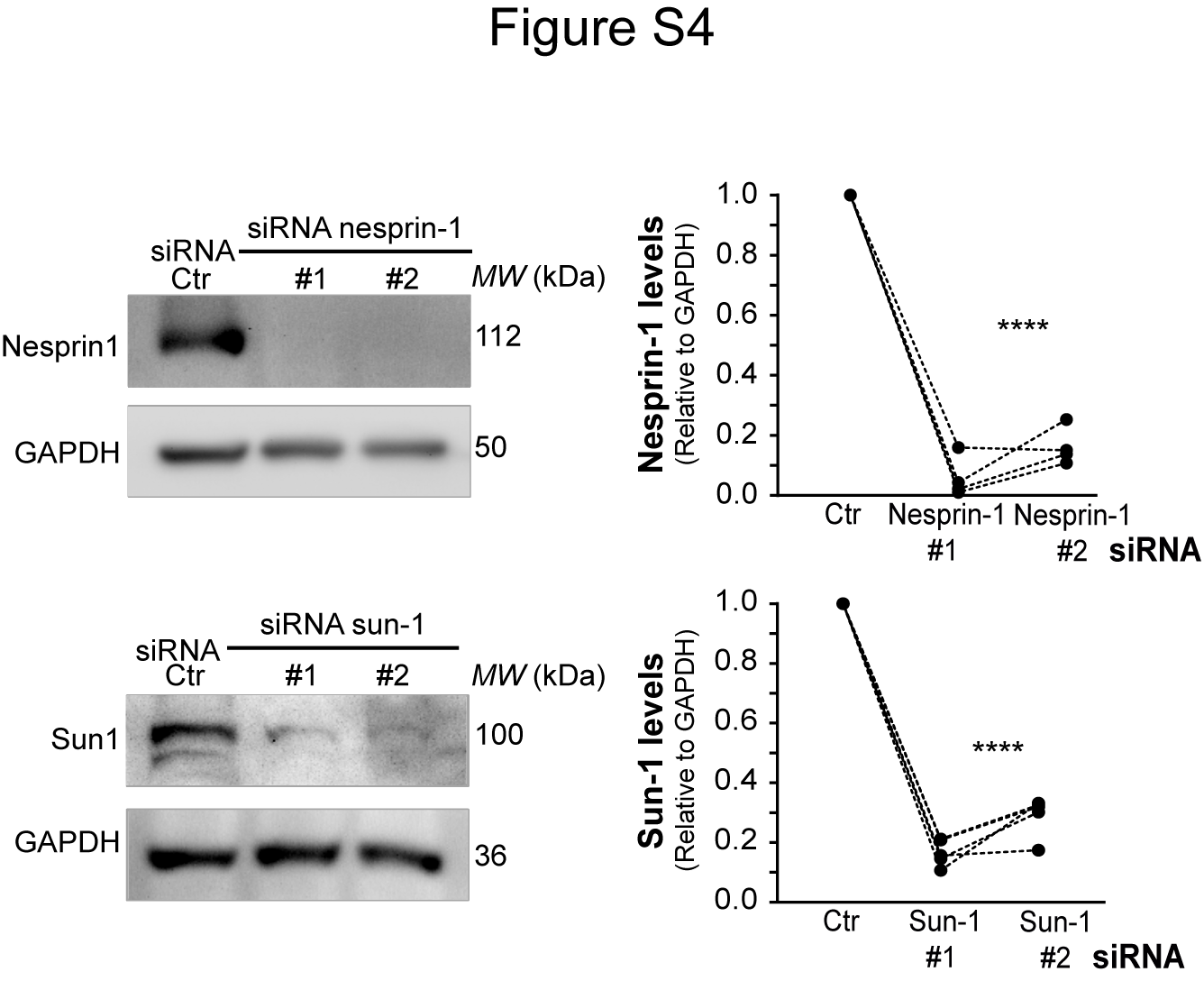

Supplement: Supplementary Figure 4 — Left: Nesprin-1 or Sun-1 protein and GAPDH levels from control or Nesprin-1 and Sun-1-silenced cells, detected by Western blot. Right: Nesprin-1 and Sun-1 levels normalized to GAPDH levels. n=4 independent experiments. Statistical analyses: ANOVA followed by Sidak’s multiple comparison tests. ****p<0.0001. [file Image_4.tif]

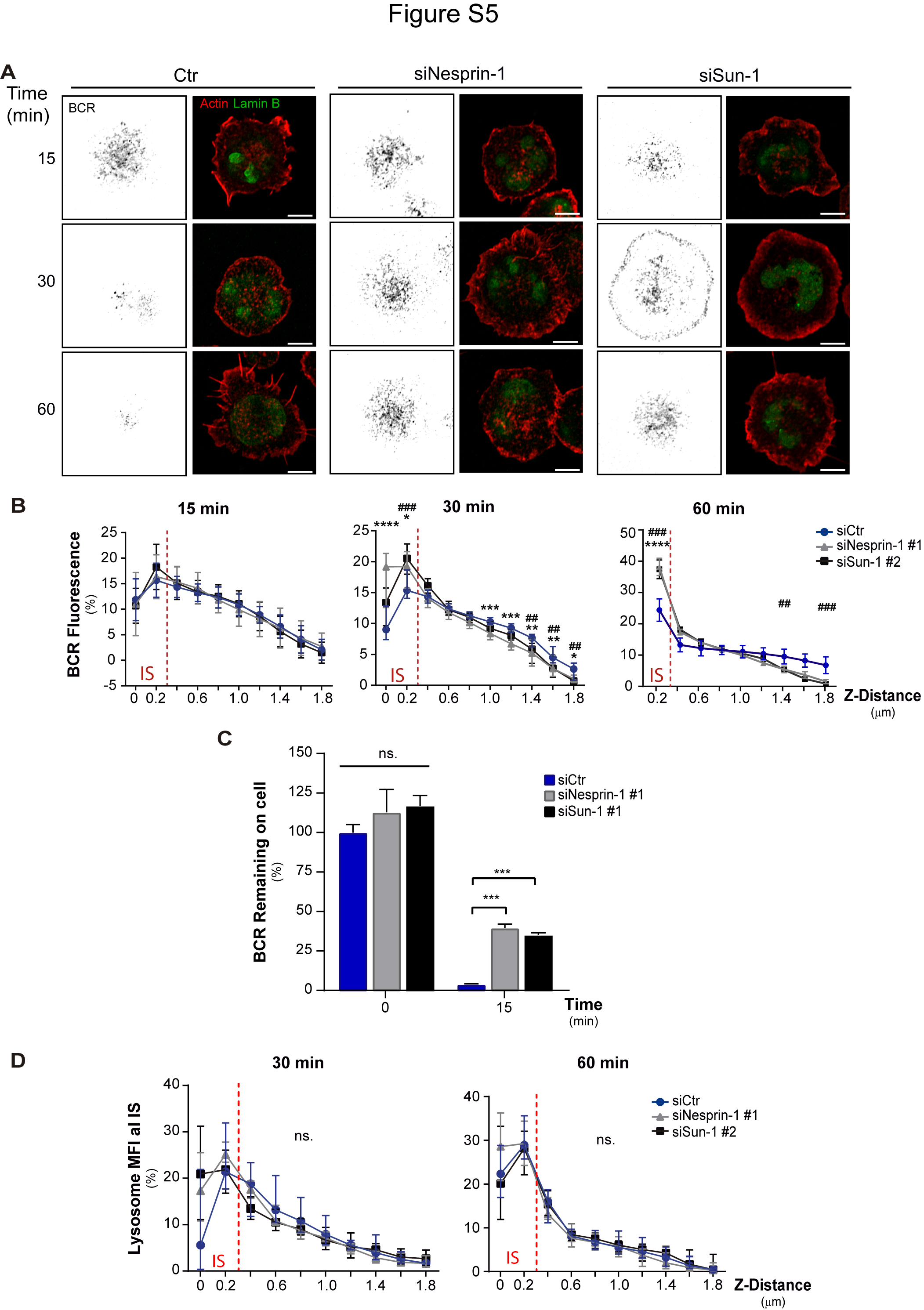

Supplement: Supplementary Figure 5 — (A) Confocal images of control, Nesprin-1- and Sun-1-silenced B cells activated on antigen-coated dishes for 15, 30, and 60 min. BCR (grey), actin (red), and Lamin B (green). Scale bar 5 µm. (B) BCR fluorescence intensity at the immune synapse (0-0.2 μm) and intracellular localization (0.4-2 μm) at each z plane. Mixed-effects analysis and Dunnett’s multiple comparisons test. n>30. * and # indicate statistical differences between control and Nesprin-1- or Sun-1-silenced cells, respectively. */# p<0.05, **/##p<0.01, ***/###p<0.001, ****/####p<0.0001. Mean and 95% CI line are shown. (C) Quantification of BCR remaining on the cell surface. Control and Nesprin-1- and Sun-1-silenced B cells (resting or activated for 15 min) were incubated with anti-mouse Alexa-647 to label the BCR in non-permeabilized conditions. Average levels measured from Control cells was considered as 100% for comparison rates. n≥60. (C) Lysosome fluorescence intensity at the immune synapse (0-0.2 μm) and intracellular localization (0.4-2 μm) according to Z distance. Cells were activated on antigen-coated dishes for 30 and 60 min. Mixed-effects analyses and Dunnett’s multiple comparisons tests confirmed no significant differences (ns). [file Image_5.tif]

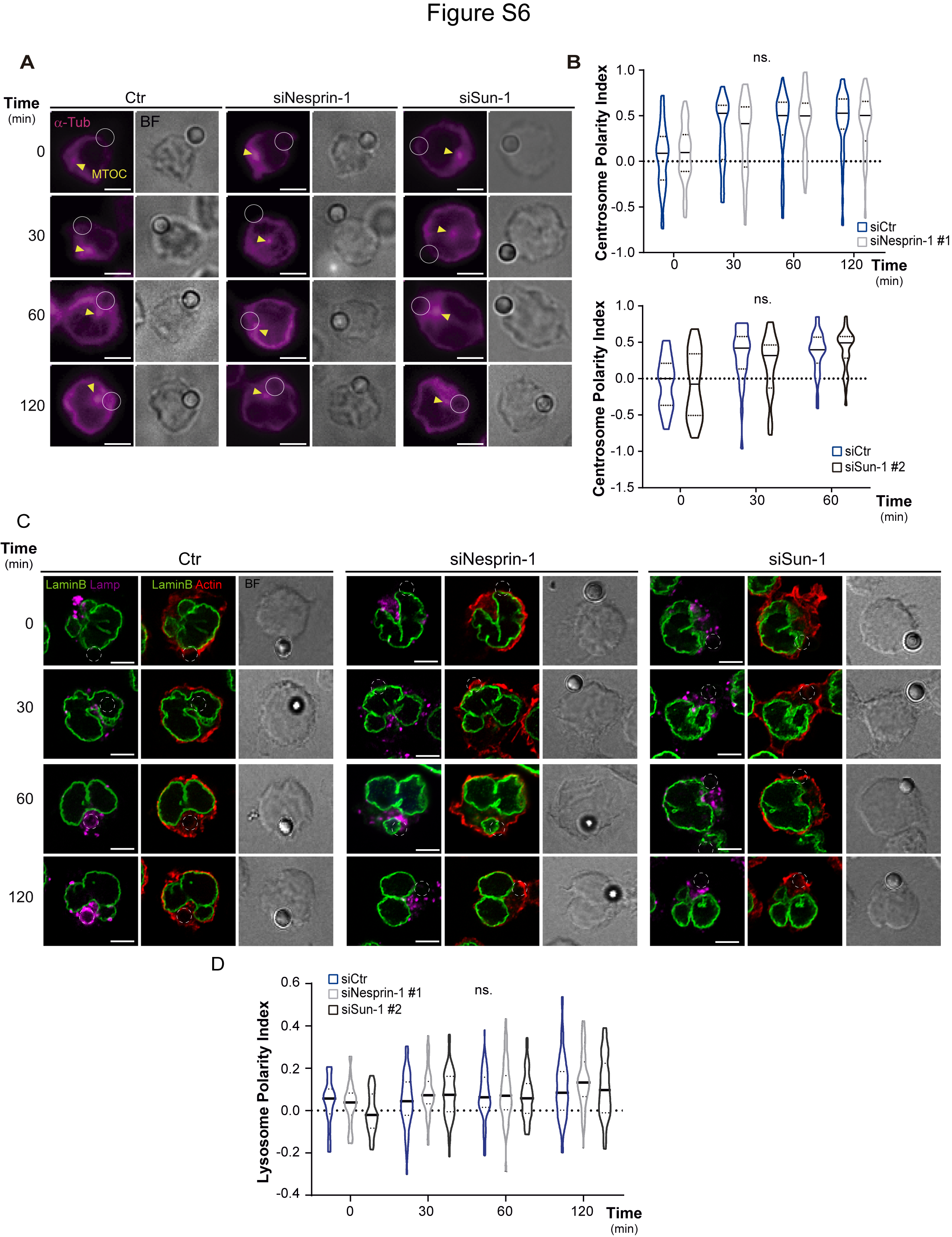

Supplement: Supplementary Figure 6 — (A, B) Representative images of microtubules (α-tubulin, magenta) and centrosome polarity indexes for control and Nesprin-1- and Sun-1-silenced B cells, activated with antigen-coated beads at indicated times; n≥70. (C) Representative confocal images of silenced B cells activated as in (A). Nucleus (Lamin B, green), actin (phalloidin, red), and lysosomes (LAMP1, magenta). Scale bar 5 µm. (D) Lysosome polarity index; n≥80. Mann-Whitney test showed no significant differences (ns) in (B) and (D). Statistical analyses: Kruskal-Wallis with Dunn’s tests; n≥40 cells from two independent experiments. *p<0.05, **p<0.01. White circles indicate bead position. [file Image_6.tif]
